# Workforce situation of the Chinese mental health care system: results from a cross-sectional study

**DOI:** 10.1186/s12888-022-04204-7

**Published:** 2022-08-22

**Authors:** Jing-Li Yue, Na Li, Jian-Yu Que, Si-Fan Hu, Na-Na Xiong, Jia-Hui Deng, Ning Ma, Si-Wei Sun, Rui Chi, Jie Shi, Hong-Qiang Sun

**Affiliations:** 1 Peking University Sixth Hospital, Peking University Institute of Mental Health, NHC Key Laboratory of Mental Health (Peking University), National Clinical Research Center for Mental Disorders (Peking University Sixth Hospital), Huayuanbei Road 51, Haidian District, Beijing, 100191 China; 2 National Institute on Drug Dependence, Peking University, Beijing, China

**Keywords:** Mental health, Workforce, Psychotherapy, Capacity, Government policy

## Abstract

**Background:**

High-quality mental health services can improve outcomes for people with mental health problems and abate the burden of mental disorders. We sought to identify the challenges the country’s mental health system currently faces and the human resource situation related to psychological services and to provide recommendations on how the mental health workforce situation could be addressed in China.

**Methods:**

This study used a cross-sectional survey design. A web-based questionnaire approach and a convenience sampling method were adopted. It was carried out from September 2020 to January 2021 in China, and we finally included 3824 participants in the analysis. Descriptive statistical analysis of the characteristics of the study sample was performed. The risk factors for competence in psychological counseling/psychotherapy were assessed using multiple linear regression analysis.

**Results:**

Workforce related to psychotherapy is scarce in China, especially in Western China and community mental health sectors. Psychiatrists (39.1%) and nurses (38.9%) were the main service providers of psychotherapy in psychiatric hospitals, and clinical psychologists (6.9%) and counsellors (5.0%) were seriously scarce in mental health care sectors. A total of 74.2% of respondents had no systematic psychological training, and 68.4 and 69.2% of them had no self-experience and professional supervision, respectively. Compared with clinical psychologists and counselors, psychiatrists and nurses had less training. Systematic psychological training (β = − 0.88), self-experience (β = − 0.59) and professional supervision (β = − 1.26) significantly influenced psychotherapy capacity (*P*<0.001).

**Conclusions:**

Sustained effort will be required to provide a high-quality, equitably distributed psychotherapy workforce in China, despite challenges for community mental health sectors and western China being likely to continue for some time. Because mental illness is implicated in so many burgeoning social ills, addressing this shortfall could have wide-ranging benefits.

**Supplementary Information:**

The online version contains supplementary material available at 10.1186/s12888-022-04204-7.

## Introduction

The global burden of mental disorders has risen in all countries, increasing by 13.5% between 2007 and 2017 [1, 2]. In 2016, mental disorders affected more than 1 billion people worldwide [3]. It was the leading cause of years lived with disability (YLDs), accounting for 32.4% [4]. It is projected that the estimated economic cost will increase to six trillion dollars by 2030 [5]. In China, most mental disorders have become more common in the past 30 years. The lifetime prevalence of mental disorders was 16.6% [6]. This disease burden reflects characteristics of mental disorders with high prevalence and high severity [7].

Despite the growing public health burden of mental disorders, there is still widespread neglect of the human workforce for mental health care in low- and middle-income countries (LMICs) [8]. China, as one of the two most populous countries in the world, has improved greatly in psychiatric human resources in the context of the World Health Organization (WHO) Comprehensive Mental Health Action Plan (2013–2020) and its National Mental Health Plan (2015–2020) [9]. However, the available evidence still highlights an alarming scarcity and inequitable distribution of professionals available, especially a severe lack of nonpsychiatric mental health professionals such as psychotherapists [9, 10]. The number of psychotherapists nationwide is only approximately 5000 [9]. Like many other LMICs, very little mental health service research has been conducted in China, although the actual extent of the workforce problem is unknown.

Mental health problems exist along a continuum from mild, time-limited distress to chronic, progressive, and severely disabling conditions. Psychotherapies, activities carried out by means of scientifically recognized procedures for the detection, cure or alleviation of mental disorders, are crucial ingredients for the treatment of mental health problems [11]. Most people with mental disorders prefer psychotherapies over pharmacological treatments when options are offered [2]. The effect sizes for psychotherapies typically range from moderate to large, and psychotherapies seem to have a greater enduring effect than pharmacological therapies [12–16]. Additionally, the strength of evidence for psychotherapies is at least as strong as for other treatment methods [2]. However, access to these therapies is very low in most people in the majority of countries, and the quality of mental health services is uneven [17]. It has been reported that 20% of depressed people receive minimally adequate treatment (meaning either pharmacotherapy with ≥ 1 month of a medication plus ≥ four visits to any type of medical doctor or psychotherapy with ≥ eight visits with any professional) in high-income countries, while only 3.7% receive minimally adequate treatment in LMICs [18, 19]. This is primarily because there are very few skilled practitioners of psychosocial therapies in most countries, and awareness of their availability is lacking [2].

A comprehensive study of the human resource situation related to psychotherapy in China’s mental health services has not yet been conducted. The human resource distribution and quality in China need to be identified and addressed before targets for strengthening the mental health system can be set for the country. The present study sought to identify challenges the country’s mental health system currently faces and provide recommendations on how the human resource situation in mental health could be addressed in China.

## Methods

### Study design, sample, and procedures

This study used a cross-sectional survey design. It was carried out from September 2020 to January 2021 in China. A web-based questionnaire approach was adopted considering epidemic prevention and control measures, and we also chose to apply a convenience sampling method. The questionnaire survey platform was called Wenjuanxing (https://www.wjx.cn/), a public service platform similar to the Qualtrics in China. We released a web page or QR code related to the questionnaire survey in the recruitment poster. Participants voluntarily completed the survey via WeChat (a social media app). Each WeChat ID was used only once to answer the questionnaire to avoid repeated fillings. Once participants completed the survey online, investigators had access to confidentially log in and download the data in Excel format locally.

Inclusion criteria were that individuals aged ≥ 18 were currently working and living in China and conducting psychological counseling or psychotherapy in hospitals on the Chinese mainland. Participants unable to complete the survey for any reason were excluded from the present study. The definition of psychotherapy (derived from healthcare security administration in China) in the study was an activity in which persons with theoretical training, practice and supervision conduct psycho diagnosis and offer systematic interventional processes to clients or patients using specific psychological methods and techniques to cure or alleviate mental distress [20].

Through the social psychological service system of China, which includes hospitals related to psychological services across the country, we collected samples from hospitals of different levels and types covering different areas of the country. In the present study, psychiatric and general hospitals with different levels, such as tertiary, secondary and community levels, were included, which were classified in accordance with the Chinese hospital classification management approach considering several aspects, including hospital infrastructure, medical service and management, technical level and efficiency, and quality and safety of clinical care [21]. A total of 4296 professionals participated in the survey after excluding participants who reported being unable to provide mental health services and time spent answering the questionnaire beyond the mean ± 3SD. Finally, we included 3824 participants in our analysis. The effective response rate was 89.0%.

### Measures

The data were collected using a self-administered questionnaire about mental health workforce and service capacity consisting of demographic characteristics, training situation and competence in psychological counseling or psychotherapy, which was established and modified based on the human resources of the WHO Assessment Instrument for Mental Health Systems (WHO-AIMS) to satisfy the need of the present research [22]. Before the massive distribution of the survey, we organized an expert consultation meeting to discuss and revise the questionnaire. We conducted a pilot study by inviting 20 staff members from our hospital to complete this survey, ensuring that all questions were easily understood.

The training situation was measured by four questions: (1) Have you ever attended any short-term psychological training programs in the past 5 years? (2) Have you ever attended any systematic or continuous psychological training programs (long-term training) in the past 5 years? (3) Have you ever attended any self experience in the past 5 years? (4) Have you ever received any professional psychological supervision in the past 5 years? The four questions were designed based on the concept that a qualified psychological workforce should have such experiences, including sufficient professional training, self-experience and professional supervision. Self-experience (also called self-reflection) refers to practitioners who undertook on-going regular personal psychotherapy for the duration of their careers to gain an understanding of the self-reflective process and a capacity for critical self-reflection to evaluate their own competence and to be aware of their strengths and limitations [23]. We used a visual analog scale ranging from 0 to 10 to assess participants’ ability to provide psychological services: Please rate your current competency in psychological counseling or psychotherapy (0: completely incompetent, 10: completely competent).

### Statistical analysis

The data were analyzed using the Statistical Package for the Social Sciences (SPSS) version 24.0 (IBM). The normal distribution of continuous variables was analyzed using the Kolmogorov–Smirnov test, which showed an abnormal distribution [24]. First, we analyzed the sample on the whole level. Then, we divided the sample into three groups by geographical region: eastern area (i.e., Beijing, Shandong, Zhejiang, etc.), central area (i.e., Shanxi, Henan, Hubei, etc.), and western area (i.e., Gansu, Sichuan, Yunnan, etc.). The zoning standard of geographical areas in the current survey was based on three economic zones in mainland China considering natural conditions, economic resources and development, science and technology, etc. A [25]. A comparison between groups was performed using the Kruskal–Wallis H test for continuous variables and the χ^2^ test for categorical variables. Post hoc testing was used to further determine the differences between the two groups. In the present study, the absolute values of the adjusted standardized residuals not less than three were considered significant differences. Variance Inflation Factor (VIF) values larger than 10 were regarded as having the presence of multicollinearity [24], and in our study, the VIF values of all independent variables were < 10. We measured Kendall’s tau-b correlation analysis among training situations and competency and further performed multiple linear regression analysis with competence in psychological counseling or psychotherapy using age, gender, hospital levels, education level, workforce types, titles, specialty and psychological training situations as predictive factors [26]. The dependent variable was competence in psychological counseling or psychotherapy. Subgroup analysis was performed to discuss the possible associations between those variables and competence in different regions. Regression coefficient estimates (β), standard error (SE) of β, 95% confidence intervals (CI) of β, and *P* values were analyzed. All tests were two-tailed with the level of statistical significance defined as *P* <  0.05 [27].

### Ethical considerations

The study was approved by the Ethical Committee of the Peking University Sixth Hospital. All subjects gave their informed consent for inclusion before participating in the survey. All procedures were made to comply with the ethical standards of the institutional or national research committee and with the 1964 Helsinki declaration and its later amendments or similar ethical standards [6].

## Results

### Inadequate distribution of psychotherapy professionals

Table 1 presents the characteristics of participants by different regions. A total of 36.6% of respondents were from the eastern area, 22.8% were from the central area and 40.6% were from the western area. Of 3824 participants (mean age = 35.62 years, SD = 8.81 years), 83.7% were less than 45 years old. A total of 69.4% of the participants were women. A total of 60.9% of people had received a bachelor’s degree, and nearly 10% had a master’s degree or higher. The workforces for psychotherapy were mainly psychiatrists and nurses (39.1 and 38.9%, respectively); however, there were fewer counselors or therapists (5.0 and 6.9%, respectively). There were significant differences in age, gender, hospital levels, education levels, titles and workforce types among the different regions (*P* <  0.05).

**Table 1:** Demographic characteristics of participants by geographical region

Characteristics	Geographical Regions				
	Total (*N* = 3824)	Eastern area (*N* = 1401)	Central area (*N* = 872)	Western area (*N* = 1551)	*P*
Workforce types N (%)					< 0.001
Psychiatrist	1495 (39.1)	736 (52.5)	262 (30.0)	497 (32.0)	
Psychotherapist	263 (6.9)	133 (9.5)	66 (7.6)	64 (4.1)	
Counselor	193 (5.0)	75 (5.4)	66 (7.6)	52 (3.4)	
Nurse	1487 (38.9)	248 (24.8)	379 (43.5)	760 (49.0)	
Other	386 (10.1)	109 (7.8)	99 (11.4)	178 (11.5)	
Age N (%)					< 0.001
≤ 45	3178 (83.7)	1136 (81.3)	739 (85.4)	1303 (84.9)	
> 45	619 (16.3)	261 (18.7)	126 (14.6)	232 (15.1)	
Gender N (%)					< 0.001
Male	1172 (30.6)	520 (37.1)	310 (35.6)	342 (22.1)	
Female	2652 (69.4)	881 (62.9)	562 (64.4)	1209 (77.9)	
Education level N (%)					< 0.001
High school or below	103 (2.7)	19 (1.4)	55 (6.3)	29 (1.9)	
Junior college	1015 (26.5)	243 (17.3)	289 (33.1)	483 (31.1)	
Bachelor	2329 (60.9)	927 (66.2)	459 (52.6)	943 (60.8)	
Master	355 (9.3)	201 (14.3)	65 (7.5)	89 (5.7)	
Doctor	22 (0.6)	11 (0.8)	4 (0.5)	7 (0.5)	
Title N (%)					< 0.001
Primary	1827 (47.8)	524 (37.4)	464 (53.2)	839 (54.1)	
Intermediate	1117 (29.2)	506 (36.1)	227 (26.0)	384 (24.8)	
• Subsenior	476 (12.4)	225 (16.1)	75 (8.6)	176 (11.3)	
Senior	137 (3.6)	69 (4.9)	23 (2.6)	45 (2.9)	
Other	267 (7.0)	77 (5.5)	83 (9.5)	107 (6.9)	
Hospital levels N (%)					< 0.001
Tertiary general hospital	315 (8.2)	59 (4.2)	70 (8.0)	186 (12.0)	
Tertiary psychiatric hospital	1640 (42.9)	454 (32.4)	337 (38.6)	859 (54.7)	
Secondary-level general hospital	394 (10.3)	164 (11.7)	126 (14.4)	104 (6.7)	
Secondary-level psychiatric hospital	1121 (29.3)	598 (42.7)	238 (27.3)	285 (18.4)	
Community hospitals	354 (9.3)	126 (9.0)	101 (11.6)	127 (8.2)	

There was a similar workforce distribution between the central and western regions, in which psychotherapies were mainly carried out by nurses, followed by psychiatrists. However, the opposite trend was observed in the eastern region. In addition, the workforce of psychotherapists or counselors accounted for nearly 15% in the eastern and central regions, almost twice as much as in the western region. People for psychotherapies in the eastern region had higher education levels than those in the central and western regions, particularly bachelor’s degrees (or above), which accounted for more than 80% of the population in the eastern region. Nearly 30–40% of people had only a junior college and high school or below education level in the central and western regions. More than 70.0% of the psychotherapies were carried out in psychiatric hospitals on the whole, and similar trends were seen in all three regions. In western China, nearly 70% of the workforce for psychotherapies was in tertiary hospitals. Fewer psychotherapy workers in the general hospitals were seen in eastern and central China (see Table 1).

### Inadequate psychotherapy training

The workforces for psychological training were severely insufficient. Overall, during the past 5 years, nearly half of the participants did not receive short-term psychological training, more than 74% did not receive systematic and continuous psychological training, and nearly 70% did not receive self-experience or psychological supervision (see Table 2).

**Table 2:** Training situation of participants by geographical regions

		Geographical Regions				
		Total (*N* = 3824)	Eastern area (*N* = 1401)	Central area (*N* = 872)	Western area (*N* = 1551)	*P*
Short-term training N (%)	Yes	1919 (50.2)	750 (53.5)	452 (51.8)	717 (46.2)	< 0.001
	No	1905 (49.8)	651 (46.5)	420 (48.2)	834 (53.8)	
Long-term training N (%)	Yes	988 (25.8)	395 (28.2)	255 (29.2)	338 (21.8)	< 0.001
	No	2836 (74.2)	1006 (71.8)	617 (70.8)	1213 (78.2)	
Self-experience N (%)	Yes	1210 (31.6)	423 (30.2)	314 (36.0)	473 (30.5)	< 0.001
	No	2614 (68.4)	978 (69.8)	558 (64.0)	1078 (69.5)	
Supervision N (%)	Yes	1177 (30.8)	460 (32.8)	302 (34.6)	415 (26.8)	< 0.001
	No	2647 (69.2)	941 (67.2)	570 (65.4)	1136 (73.2)	

Compared with other workforce types, psychotherapists and counselors had more psychological training. Taking the psychotherapist as an example, nearly 80% received short-term training, 50% received continuous psychological training, more than 40% received self-experience and nearly 65% received psychological supervision in the past 5 years. Nearly 70% of psychiatrists and nurses did not receive continuous psychological training, self-experience or psychological supervision in the past 5 years (see Table S1).

For short-term training, almost half of the people took part in the training in the past 5 years at different hospital levels. Generally, the majority of people in all hospitals did not receive any long-term training, self-experience or supervision in the past 5 years. Less continuous training was seen in psychiatric and community hospitals. People in the community hospitals had less self-experience and psychological supervision (see Table S2).

### Insufficient capacity in psychotherapy of mental health workers

Competence in psychological counseling or psychotherapy among different regions is indicated as the median and quartiles in Table 3, and significant regional differences were seen (*P* <  0.05). The competence of respondents from the eastern and central regions was higher than that of respondents from the western region. Psychotherapists and counselors had higher competence than psychiatrists and nurses (see Table S3). Respondents from community hospitals had lower competency than respondents from other hospital types. The details are provided in the supplementary materials (see Table S4).

**Table 3:** Competence in psychological counseling/psychotherapy by geographical region

Variable	Geographical Regions			
	Eastern area (*N* = 1401)	Central area (*N* = 872)	Western area (*N* = 1551)	*P*
Psychological competency *median (Q1, Q3)*	6 (5,8)	6 (4,8)	5 (3,7)	< 0.001

### Multiple linear regression analysis for counseling and psychotherapy capacity

We first performed Kendall’s tau-b correlation analysis, which indicated that competency was negatively associated with psychotherapy training, self-experience and supervision alone (*r*_short-term training_ = − 0.22, *P* <  0.001; *r*_long-term training_ = − 0.28, *P* <  0.001; *r*_self-experience_ = − 0.24, *P* <  0.001; *r*_supervision_ = − 0.32, *P* <  0.001, respectively) (see Table S5). The results of the multiple linear regression analysis of factors influencing competence scores are shown in Table 4. After adjusting for all covariates, the training situation variables still showed significant results in model 2, with an adjusted R^2^ value of 0.19. Attending short-term training, long-term training, self-experience and receiving professional psychological supervision predicted a high level of competence in psychological counseling or psychotherapy (F = 83.66, *P* <  0.001). The effects of variables on the variance in competence scores are displayed. Training situations explained 16.0% of the variance in competence scores. Demographic variables accounted for an additional 3.3% of the variance in competence scores. The results of subgroup analyses were similar to those of the general multiple linear regression analysis. However, in the central area and western area, there was no statistically significant association between attending short-term training and competence in counseling or psychotherapy. The other three types of training situations were all significant predictive variables among different regions (see Table 5).

**Table 4:** Multiple linear regression analysis of counseling and psychotherapy competence

	Model 1	Model 2
Short-term training (Yes/No)	−0.327^**^	−0.223^*^
Long-term training (Yes/No)	−0.887^***^	−0.884^***^
Self-experience (Yes/No)	− 0.460^***^	− 0.586^***^
Supervision (Yes/No)	−1.362^***^	−1.260^**^
Gender (Male/female)		−0.284^**^
Age (≤ 45/> 45)		0.293^*^
Geographical regions		−0.289^**^
Hospital levels		−0.236^***^
Education level		−0.071
Workforce		−0.190^***^
Title		−0.028
ΔR^2^	0.161	0.196
Adjusted R^2^	0.160	0.193
*F*	182.102^***^	83.660^***^

^*^P <  0.05, ^**^P < 0.01, ^***^P < 0.001; In step 1, training situations were added. In step 2, demographics were added

**Table 5:** Subgroup analyses stratified by geographical regions

Variables	Geographical Regions								
	Eastern area			Central area			Western area		
	β (SE)	95% CI of β	*P*	β (SE)	95% CI of β	*P*	β (SE)	95% CI of β	*P*
Short-term training	−0.409 (0.154)	−0.711 ~ − 0.108	< 0.01	−0.186 (0.209)	− 0.596 ~ 0.224	0.374	− 0.045 (0.160)	−0.360 ~ 0.269	0.777
Long-term training	−0.898 (0.185)	− 1.261 ~ − 0.536	< 0.001	− 0.591 (0.244)	−1.070 ~ − 0.112	< 0.05	−1.010 (0.202)	− 1.407 ~ − 0.613	< 0.001
Self-experience	−0.350 (0.167)	− 0.677 ~ − 0.023	< 0.05	−0.715 (0.232)	−1.171 ~ − 0.259	< 0.01	−0.741 (0.174)	−1.083 ~ − 0.400	< 0.001
Supervision	−1.068 (0.178)	− 1.418 ~ − 0.719	< 0.001	− 1.322 (0.244)	−1.800 ~ − 0.844	< 0.001	−1.363 (0.195)	− 1.745 ~ − 0.980	< 0.001
Gender	−0.462 (0.135)	− 0.726 ~ − 0.198	< 0.01	−0.022 (0.183)	− 0.381 ~ 0.338	0.906	− 0.354 (0.167)	−0.681 ~ − 0.026	< 0.05
Age	0.461 (0.173)	0.121 ~ 0.800	< 0.01	0.386 (0.254)	−0.112 ~ 0.885	0.129	−0.034 (0.202)	−0.430 ~ 0.362	0.867
Hospital levels	−0.279 (0.060)	−0.397 ~ − 0.161	< 0.001	−0.118 (0.075)	− 0.265 ~ 0.029	0.115	− 0.262 (0.060)	−0.379 ~ − 0.145	< 0.001
Education level	0.132 (0.114)	−0.091 ~ 0.356	0.245	0.160 (0.137)	−0.108 ~ 0.428	0.241	−0.445 (0.123)	−0.686 ~ − 0.205	< 0.001
Workforce	0.006 (0.048)	−0.087 ~ 0.099	0.896	−0.265 (0.067)	−0.396 ~ − 0.134	< 0.001	−0.321 (0.050)	− 0.420 ~ − 0.222	< 0.001
Title	−0.007 (0.061)	− 0.127 ~ 0.113	0.913	− 0.137 (0.071)	−0.275 ~ 0.002	0.054	0.042 (0.062)	−0.079 ~ 0.163	0.493
ΔR^2^	0.188			0.201			0.198		
Adjusted R^2^	0.182			0.192			0.192		
*F*	32.067^***^			21.496^***^			37.543^***^		

*P<0.05, **P<0.01,***P<0.001

## Discussion

This study has painted a picture of the psychotherapy human resource situation in China. The results revealed an alarming scarcity of trained psychotherapy professionals within the mental health care sectors in China. To the authors’ knowledge, this is the first study that has attempted to assess the mental health human resource situation focusing on psychotherapy in China.

The health workforce is foundational to high-quality mental health services, thus calling for improvements in health through sustainable expansion of the health workforce [28]. Psychiatrist workforce development efforts in China have represented concrete steps; however, other professional workforces related to psychotherapy are deeply underdeveloped and still unclear in China. Data from the present study indicated that in eastern and central China, psychiatrists and nurses (nearly accounting for 1/4, respectively) were the main service providers of psychotherapy in psychiatric hospitals, who are usually not initially qualified as psychological professionals and rarely have psychological training. Clinical psychologists (6.9%) are seriously scarce in mental health care sectors in China. In Italy, there were 2/3 psychologists and 1/3 medical doctors among the psychotherapy workforce in 2014. Only psychologists and medical doctors who have a minimum of 4 years of postgraduate training can practice in Italy [29]. Surprisingly, nearly 2/5 of the psychotherapy workforce in central and western China had only junior college and high school or below education levels. The minimum requirements on education and training for psychotherapy workforce (such as at least being graduates of medicine or psychology) are in need of specialization in psychotherapy in China.

Challenges in the quality and training of existing personnel are also concerns, and in-service psychotherapy training is a highly neglected area in China. The results from this study indicated that long-term psychological training and professional supervision, which are dire shortages in China, significantly influenced psychotherapy capacity. The crisis of the health sector has not gone unnoticed by the National Health Commission in China. The government issued guidance on strengthening mental health services and set up a social psychological service system in 2016, highlighting key human resource constraints and identifying strategies to address these constraints [30]. Despite the promise of these initiatives, based on the findings from this study, it seems that the effects of these initiatives have not adequately been felt in the mental health sectors. The government not only needs to increase the size of the mental health workforce but also strengthen the training of psychotherapy to enhance the quality of mental health services.

Long-term continuous training and psychological supervision have been found to greatly impact capacity. Taking the German situation of psychotherapy as an example, psychotherapeutic training specifically focused on the following elements: (1) 620 hours of theoretical lessons; (2) 120 hours of self-experience; (3) 1200 hours of practicum in a psychiatric institution; and (4) 600 hours of practicum in a different psychiatric or psychotherapeutic/outpatient institution under 150 hours of supervision [31]. The measurable and minimum core criteria of psychotherapeutic training about theoretical training, self-experience and supervision should be created locally in China and similar countries. Innovative strategies can facilitate local shortages. For example, digital applications can extend the capacity and reach of the limited number of mental health specialists by facilitating off­site supervision and mentoring of local health and lay providers. Online training and the use of peers to supervise therapy quality with structured scales and feedback also addressed these barriers [32].

Staff shortages are serious in community hospitals, and mental health service provision needs to be strengthened in all community and primary health­care platforms in western China. Failure to properly address the human resource crisis in mental health has led to poor service delivery and feelings of incompetence among staff and an overreliance on psychiatric hospitals rather than community psychiatric services. These problems appear to be common consequences of inadequate human resources for mental health in other LMICs. There is a concept called task sharing, which refers to the transfer of some mental health care responsibilities from more specialized to less specialized staff [2]. Previous studies indicated that the exponential expansion of the range of providers with specific training in these psychological therapies has somewhat reduced the treatment gap for common mental disorders. It is relevant to community hospitals or hospitals in western China in a short time.

We think that two potential problems block the high-quality development of psychotherapy to a certain degree. First, there is currently a lack of authorized institutions or councils of accreditation for psychotherapy or counseling in China, which greatly restricts the development of psychological workforces on the whole society [9]. Second, clinical psychologists who own psychotherapeutic training are not allowed to conduct psychotherapies by the majority hospitals due to their insufficiency in medical experience, which hinders numerous psychology graduates from entering hospitals in China. Although a person can be a psychotherapist through an exam under the charge of the national health commission, one of the preconditions is that they must work in a health care facility. That is why nurses were the main providers of psychotherapy in China. Nurses work in hospitals and have chances or been allowed to attend the psychotherapist exam under the charge of the national health commission. The displacement of professionals reduced the capacity and quality of mental health services. Breaking down current policy barriers and establishing novel policy and regulations nationally and locally are imminent for China to be capable of coping with the high demand for mental health services, especially under COVID-19. The education and trainings vital for qualified psychotherapists or psychotherapeutic counsellors and policy implications derived from this study (recommendations summarized in Table 6) may also apply to other LMICs similar to the Chinese situation in psychotherapy.

**Table 6:** Summary of recommendations

Summary of recommendations	
a Recommendations for areas (or hospitals) of deficient workforce like western China (or community hospitals):	
a1. Learning task sharing to transfer some mental health care responsibilities from more-specialized to less-specialized staff	
b Recommendations for areas (or hospitals) like eastern China (or tertiary hospitals):	
b1. The minimum requirements on education and training for psychotherapy workforce (such as at least being graduates of medicine or psychology) are in need to make the specialization in psychotherapy	
b2. The measurable and minimum core criteria of psychotherapeutic training about theoretical training, self-experience and supervision should be created locally	
c Recommendations for policy or regulations:	
c1. The government not only needs to increase the number of mental health workforce but also strengthen the training of psychotherapy to enhance the quality of mental health services	
c2. Providing standard clinical training for psychologists and adjusting the policy to making them access to hospital more flexibly could have great potentials to expand psychotherapy-related human resources	
c3. Breaking down current policy barriers and establishing novel policy and regulations nationally and locally are imminent for China	
d Other potential recommendations:	
d1. Digital applications can extend the capacity and reach of the limited number of mental health specialists by facilitating off­site supervision and mentoring of local health and lay providers, reducing regional and hospital imbalances	
d2. Online training and the use of peers to supervise therapy quality with structured scales and feedback have a potential for alleviating shortage of experienced trainers	

Several limitations of this study should be noted. First, we used a cross-sectional design and a convenience sampling method. Although the sample covered three major regions of the country, the sample size in some provinces is still small, which limited our ability to find more specific characteristics of different regions and to generalize the results to similar countries. Second, quantitative research was employed in the study, and more detailed information might be missing; thus, a combination of quantitative and qualitative research is needed in the future. Third, the questionnaire in the present study was established and modified based on the WHO-AIMS [22, 33]. More structured tools and models should be applied to estimate the mental health workforce.

## Conclusions

Sustained effort will be required to provide a high-quality, equitably distributed psychotherapy mental health workforce in China. Although these are very promising policies and programs, challenges for community mental health sectors and western China are likely to continue for some time. If China was able to address its unmeet mental health workforce needs, this will have substantial implications, not only for a large portion of the worldwide population but also as an inspiration for other LMICs.

## Supplementary Information

**Additional file 1: Table S1**. Training Situation of Participants by Different Workforces. **Table S2**. Training Situation of Participants by Different Hospital Levels. **Table S3**. Competence in Psychological Counseling/Psychotherapy by Different Workforces. **Table S4**. Competence in Psychological Counseling/Psychotherapy by Different Hospital Levels. **Table S5**. Correlations among training situations and competency.

## Data Availability

The datasets generated during the current study are available from the corresponding author on reasonable request.

## Abbreviations

YLDs: Years Lived with Disability
LMICs: Low- and Middle-Income Countries
WHO: World Health Organization
SPSS: Statistical Package for the Social Sciences
VIF: Variance Inflation Factor
WHO-AIMS: WHO Assessment Instrument for Mental Health Systems
