# Supplementary material for: Workforce situation of the Chinese mental health care system: results from a cross-sectional study
Source: BMC Psychiatry. 2022 Aug 22;22:562. doi: 10.1186/s12888-022-04204-7 (PMC9394058; doi:10.1186/s12888-022-04204-7)
Supplement: Supplementary file 1 — Additional file 1: Table S1. Training Situation of Participants by Different Workforces. Table S2. Training Situation of Participants by Different Hospital Levels. Table S3. Competence in Psychological Counseling/Psychotherapy by Different Workforces. Table S4. Competence in Psychological Counseling/Psychotherapy by Different Hospital Levels. Table S5. Correlations among training situations and competency. [file 12888_2022_4204_MOESM1_ESM.docx]

**BMC Psychiatry**

**Supplementary online material**

Workforce situation of the Chinese mental health care system: results from a cross-sectional study

Jing-Li Yue^1#^, Na Li^1#^, Jian-Yu Que^1^, Si-Fan Hu^1^, Na-Na Xiong^1^, Jia-Hui Deng^1^, Ning Ma^1^,

Si-Wei Sun^1^, Rui Chi^1^, Jie Shi^2^, Hong-Qiang Sun^1*^

^1^Peking University Sixth Hospital, Peking University Institute of Mental Health, NHC Key Laboratory of Mental Health (Peking University), National Clinical Research Center for Mental Disorders (Peking University Sixth Hospital), Beijing, China

^2^National Institute on Drug Dependence, Peking University, Beijing, China

^#^ The authors contributed equally to this work.

^*^ Corresponding author:

Prof. Hong-Qiang Sun, MD, PhD

Email: sunhq@bjmu.edu.cn

Peking University Sixth Hospital, Peking University Institute of Mental Health, NHC Key Laboratory of Mental Health (Peking University), National Clinical Research Center for Mental Disorders (Peking University Sixth Hospital), Huayuanbei Road 51, Haidian District, 100191, Beijing, China.

**Table S1.** Training Situation of Participants by Different Workforces

|  |  | | | Workforces | | | |  | | |
| --- | --- | --- | --- | --- | --- | --- | --- | --- | --- | --- |
|  |  | Total  (N=3824) | Psychiatrist (N=1495) | | Psychotherapist (N=263) | Counselor (N=193) | Nurse (N=1487) | | Other  (N=386) | *P* |
| Short-term training N (%) | Yes | 1919 (50.2) | 820 (54.8) | | 204 (77.6) | 130 (67.4) | 593 (39.9) | | 172 (44.6) | < 0.001 |
|  | No | 1905 (49.8) | 675 (45.2) | | 59 (22.4) | 63 (32.6) | 894 (60.1) | | 214 (55.4) |  |
| Long-term training N (%) | Yes | 988 (25.8) | 365 (24.4) | | 130 (49.4) | 67 (34.7) | 346 (23.3) | | 80 (20.7) | < 0.001 |
|  | No | 2836 (74.2) | 1130 (75.6) | | 133 (50.6) | 126 (65.3) | 1141 (76.7) | | 306 (79.3) |  |
| Self-experience N (%) | Yes | 1210 (31.6) | 384 (25.7) | | 115 (43.7) | 94 (48.7) | 506 (34.0) | | 111 (28.8) | < 0.001 |
|  | No | 2614 (68.4) | 1111 (74.3) | | 148 (56.3) | 99 (51.3) | 981 (66.0) | | 275 (71.2) |  |
| Supervision N (%) | Yes | 1177 (30.8) | 427 (28.6) | | 170 (64.6) | 101 (52.3) | 395 (26.6) | | 84 (21.8) | < 0.001 |
|  | No | 2647 (69.2) | 1068 (71.4) | | 93 (35.4) | 92 (47.7) | 1092 (73.4) | | 302 (78.2) |  |

**Table S2.** Training Situation of Participants by Different Hospital Levels

|  | Hospital Levels | | | | | | |
| --- | --- | --- | --- | --- | --- | --- | --- |
|  | Total  (N=3824) | Tertiary general hospital (N=315) | Tertiary psychiatric hospital (N=1640) | Secondary-level general hospital (N=394) | Secondary-level psychiatric hospital (N=1121) | Community hospitals (N=354) | *P* |
| Short-term training N (%) |  |  |  |  |  |  | < 0.001 |
| Yes | 1919(50.2) | 162 (51.4) | 779 (47.5) | 216 (54.8) | 583 (52.0) | 179 (50.6) |  |
| No | 1905(49.8) | 153 (48.6) | 861 (52.5) | 178 (45.2) | 538 (48.0) | 175 (49.4) |  |
| Long-term training N (%) |  |  |  |  |  |  | < 0.001 |
| Yes | 988(25.8) | 95 (30.2) | 392 (23.9) | 121 (30.7) | 304 (27.1) | 76 (21.5) |  |
| - No | 2836(74.2) | 220 (69.8) | 1248 (76.1) | 273 (69.3) | 817 (72.9) | 278 (78.5) |  |
| Self-experience N (%) |  |  |  |  |  |  | < 0.001 |
| Yes | 1210(31.6) | 119 (37.8) | 478 (29.1) | 139 (35.3) | 372 (33.2) | 102 (28.8) |  |
| No | 2614(68.4) | 196 (62.2) | 1162 (70.9) | 255 (64.7) | 749 (66.8) | 252 (71.2) |  |
| Supervision N (%) |  |  |  |  |  |  | < 0.001 |
| Yes | 1177(30.8) | 112 (35.6) | 493 (30.1) | 116 (29.4) | 374 (33.4) | 82 (23.2) |  |
| No | 2647(69.2) | 203 (64.4) | 1147 (69.9) | 278 (70.6) | 747 (66.6) | 272 (76.8) |  |

**Table S3.** Competence in Psychological Counseling/Psychotherapy by Different Workforces

| Variable | Workforces | | | | | |
| --- | --- | --- | --- | --- | --- | --- |
|  | Psychiatrist | Psychotherapist | Counselor | Nurse | Other | *P* |
| Psychological competency *median (Q1, Q3)* | 6 (4,7) | 7 (6,8) | 7 (5,8) | 5 (3,7) | 4 (1,6) | <0.001 |
|  | | | | | | |

**Table S4.** Competence in Psychological Counseling/Psychotherapy by Different Hospital Levels

| Variable | Hospital levels | | | | | |
| --- | --- | --- | --- | --- | --- | --- |
|  | Tertiary general hospital (N=315) | Tertiary psychiatric hospital (N=1640) | Secondary-level general hospital (N=394) | Secondary-level psychiatric hospital (N=1121) | Community hospitals (N=354) | *P* |
| Psychological competency *median (Q1, Q3)* | 6 (5,8) | 6 (4,7) | 6 (4,8) | 6 (4,7) | 4 (1,6) | <0.001 |

**Table S5. Correlations among training situations and competency**

|  | **Short-term training** | **Long-term training** | **Self-experience** | **Supervision** | **Competency** |
| --- | --- | --- | --- | --- | --- |
| **Short-term training** | **1.00** |  |  |  |  |
| **Long-term training** | **0.50^***^** | **1.00** |  |  |  |
| **Self-experience** | **0.30^***^** | **0.42^***^** | **1.00** |  |  |
| **Supervision** | **0.43^***^** | **0.53^***^** | **0.54^***^** | **1.00** |  |
| **Competency** | **-0.22^***^** | **-0.28^***^** | **-0.24^***^** | **-0.32^***^** | **1.00** |

**^*^ significant at the 0.05 level (2-tailed), ^**^ significant at the 0.01 level (2-tailed), ^***^ significant at the 0.001 level (2-tailed).**
